# Serum cystatin C is an independent biomarker associated with the renal resistive index in patients with chronic kidney disease

**DOI:** 10.1371/journal.pone.0193695

**Published:** 2018-03-07

**Authors:** Ayu Ogawa-Akiyama, Hitoshi Sugiyama, Masashi Kitagawa, Keiko Tanaka, Akifumi Onishi, Toshio Yamanari, Hiroshi Morinaga, Haruhito Adam Uchida, Kazufumi Nakamura, Hiroshi Ito, Jun Wada

**Affiliations:** 1 Department of Nephrology, Rheumatology, Endocrinology and Metabolism, Okayama University Graduate School of Medicine, Dentistry and Pharmaceutical Sciences, Okayama, Japan; 2 Department of Human Resource Development of Dialysis Therapy for Kidney Disease, Okayama University Graduate School of Medicine, Dentistry and Pharmaceutical Sciences, Okayama, Japan; 3 Department of Chronic Kidney Disease and Cardiovascular Disease, Okayama University Graduate School of Medicine, Dentistry and Pharmaceutical Sciences, Okayama, Japan; 4 Department of Cardiovascular Medicine, Okayama University Graduate School of Medicine, Dentistry and Pharmaceutical Sciences, Okayama, Japan; Baker IDI Heart and Diabetes Institute, AUSTRALIA

## Abstract

Cystatin C is a cysteine protease inhibitor that is produced by nearly all human cells. The serum level of cystatin C is a stronger predictor of the renal outcome and the risk of cardiovascular events than the creatinine level. The resistive index (RI) on renal Doppler ultrasonography is a good indicator of vascular resistance as well as the renal outcomes in patients with chronic kidney disease (CKD). However, it is unclear whether serum cystatin C is associated with signs of vascular dysfunction, such as the renal RI. We measured the serum cystatin C levels in 101 CKD patients and investigated the relationships between cystatin C and markers of vascular dysfunction, including the renal RI, ankle-brachial pulse wave velocity (baPWV), intima-media thickness (IMT), and cardiac function. The renal RI was significantly correlated with the serum cystatin C level (p < 0.0001, r = 0.6920). The serum cystatin C level was found to be a significant determinant of the renal RI (p < 0.0001), but not the baPWV, in a multivariate regression analysis. The multivariate odds ratio of the serum cystatin C level for a renal RI of more than 0.66 was statistically significant (2.92, p = 0.0106). The area under the receiver-operating characteristic curve comparing the sensitivity and specificity of cystatin C for predicting an RI of more than 0.66 was 0.882 (cutoff value: 2.04 mg/L). In conclusion, the serum cystatin C level is an independent biomarker associated with the renal RI in patients with CKD.

## Introduction

Cystatin C is a non-glycosylated 13-kD protein that is a cysteine protease inhibitor. It is a member of the human cysteine superfamily and is stably produced by all human nucleated cells [1, 2]. The serum cystatin C level has no association with age, sex, and muscle mass; thus, it has been hypothesized that the serum cystatin C level is a superior marker of the glomerular filtration rate (GFR) to the serum creatinine level[3, 4]. Regarding cardiovascular events, cystatin C has reported to be a strong predictor of the risk of all-cause mortality and cardiovascular events [1, 5]. Furthermore, in patients with hypertension, cystatin C is related to the left ventricular mass and could be a marker of cardiac hypertrophy [6].

Renal Doppler ultrasonography is a noninvasive method of obtaining the information of vascular dynamics in various renal diseases. While the diagnostic cogency of Renal Doppler ultrasonography in renal parenchymal disease (in comparison to percutaneous renal biopsy) is still under debate, recent studies have shown that the renal resistive index (RI) is correlated with tubulointerstitial lesions and vascular lesions in the kidney [7–10]. The renal RI is a simple parameter that is calculated as follows: [(peak systolic velocity–end diastolic velocity)/peak systolic velocity] [11]. Previous reports have shown that the renal RI is associated with the renal prognosis [9, 12–14]. The RI is thought to be a good indicator of renal vascular resistance and an increased renal RI as evaluated by the pulse wave velocity [15], the common carotid intima-media thickness [16], and the diurnal change of blood pressure [17] is associated with systemic atherosclerosis. Furthermore, the renal RI has reported to be a predictor of cardiovascular events [18, 19].

We hypothesized that serum cystatin C is a significant biomarker associated with the renal RI. Thus, this study investigated the relationships between the serum cystatin C level and markers of vascular dysfunction, including the renal RI, ankle-brachial pulse wave velocity (baPWV), intima-media thickness (IMT), and the cardiac function in human subjects with CKD.

## Methods

### Subjects

Our study includes patients who were admitted to the Renal Unit of Okayama University Hospital and that of Kochi Medical Center. We diagnosed all of the patients with CKD based on the National Kidney Foundation K/DOQI guidelines [20]. We carried out all procedures in the present study according to institutional and national ethical guidelines for human studies and the guidelines outlined in the Declaration of Helsinki. This study was approved by the ethics committee of Okayama University Graduate School of Medicine, Dentistry and Pharmaceutical Sciences (No. 1063 and 1585). We obtained written informed consent from all subjects. This study was recorded with the Clinical Trial Registry of the University Hospital Medical Information Network (registration number UMIN 000014329).

### Laboratory measurements

We ran blood examination of all subjects under standardized conditions. The creatinine, hemoglobin, total cholesterol, low-density lipoprotein (LDL) cholesterol, high-density lipoprotein (HDL) cholesterol, protein, albumin, calcium, phosphate, uric acid, HbA1c and BNP levels were quantified by the standard techniques. A 24-h urine sample was collected to determine albuminuria. We calculated the eGFR according to the simplified version of the Modification of Diet in Renal Disease (MDRD) formula [eGFR = 194× (sCr)^-1.094^× (age)^-0.287^(if female×0.739)] [21]. The serum cystatin C level was quantified by an immunologic turbid metric assay (Nescoat GC Cystatin C; Alfresa Pharma, Osaka, Japan) [22], and the serum intact PTH levels were measured using an electro chemiluminescence immunoassay (LSI Medience Corporation, Tokyo, Japan).

### Vascular assessments

#### Measurement of the resistive index (RI)

We examined Ultrasonic Pulsed Doppler to measure the intra-renal arterial circulation using a 2.5-MHz sector transducer (SSD-5500; Aloka, Tokyo, Japan). The renal length was defined as the maximum longitudinal axis. Intra-renal Doppler signals were received from the interlobar arteries at the corticomedullary junction. We examined the peak systolic velocity (Vmax) and the minimal diastolic velocity (Vmin) and calculated the RI (peak systolic velocity minus minimum diastolic velocity/peak systolic velocity) using four measurements (two from each kidney). Measurements were performed by two expert nephrologists who had been blinded to all other information concerning the subjects. We defined the independent risk factors for the progression of CKD in accordance with a previous study that showed that the median RI was a predictor of the cardiovascular and renal outcomes [18]. Since no statistically significant differences were recognized in the RI values of the right and left kidneys, we used the mean RI of the right and left kidneys for the subsequent analyses.

#### Measurement of the ankle-brachial pulse wave velocity (baPWV)

Pulse wave velocity (PWV) measurements were obtained using an automatic device (FORM/ABI; Colin, Komaki, Japan) after the subjects had lain at rest in their beds for at least five minutes, as previously described [23, 24]. This device provides recording of the baPWV and the brachial and ankle BPs on both sides simultaneously. We determined that the subjects with a baPWV of ≥ 1400 had arterial sclerosis, since a baPWV of ≥ 1400 cm/s has been reported to be an independent predictor of the risk of atherosclerotic cardiovascular disease [25]. The office blood pressure was also measured.

#### Measurement of the intima–media thickness

We performed ultrasonography of the carotid artery using a high-resolution real-time scanner with a 7.5-MHz transducer. The measurement of the carotid IMT on both side was conducted in the supine position. We scanned the carotid artery and defined the thickest point as maximum IMT value in the longitudinal and transverse directions, as previously described in detail [23, 24]. We designated those subjects with an IMT of ≥1.1 mm to have atherosclerosis based on atheromatous plaques, since a previous study showed that the normal limit of IMT is ≤1.0 mm [26].

#### Echocardiography

We performed Echocardiographic studies by a cardiac ultrasound unit with a 2- to 3.5-MHz sector transducer, as previously described [27]. Measurements were performed by echocardiologists who had been blinded to all other information concerning the subjects. Standard cardiac echography of the two-dimensional parasternal long-axis and apical four-chamber views was performed in all patients, with images taken based on the guidelines of the American Society of Echocardiography [28]. The ejection fraction (EF) and the early peak diastolic annular velocity of mitral valves (e’) were measured. Diastolic dysfunction was defined as e’< 8 cm/s according to the definition of a previous study [29].

### Statistical analyses

We expressed non-continuous variables as the median (interquartile range) and continuous variables as the mean ± standard deviation (SD), as appropriate. If P values were < 0.05, the difference was considered statistically significant. The differences between groups were analyzed using Student’s *t*-test and the Mann-Whitney U-test, as appropriate. A multivariable logistic regression analysis with a simultaneous procedure was performed to identify the independent risk factors for RI elevation. We presented the P values, odds ratios (ORs) and corresponding 2-sided 95% confidence intervals (CIs) for the predictors. A receiver operating characteristic (ROC) curve analysis was performed to confirm the diagnostic efficacy of the variables, and the area under the curve (AUC) was calculated. We performed the statistical analyses using the JMP software program (version 11; SAS Institute Inc., Cary, NC, USA).

## Results

### Patient characteristics

Table 1 shows the baseline characteristics of the study population. One hundred one CKD patients with a median age of 57.0 (42.0–68.8) years were recruited in the study. The causes of CKD included glomerulonephritis (n = 48; 47.5%, biopsy proven = 42, clinical diagnosis = 6), nephrosclerosis (n = 25; 24.8%), diabetic nephropathy (n = 12; 11.9%) and “other” (n = 16; 15.8%). Of the total 101 patients, 69 patients were on antihypertensive therapy (54 patients were being treated with angiotensin receptors [ARBs] or angiotensin converting enzyme inhibitors [ACEIs], and 53 were treated with calcium channel antagonists). The median RI was 0.66 (interquartile range of 0.61–0.73). The RI, cystatin C, albuminuria, BNP and baPWV values in late-stage patients were higher than those in early- and mild-stage patients (p < 0.0001). Since the underlying causes vary for various CKD including glomerulonephritis, nephrosclerosis, and diabetic nephropathy, we further analyzed the data separating the groups based on various CKD conditions (S1 Table). The RI value in patients with diabetic nephropathy was higher than that in patients with glomerulonephritis or nephrosclerosis. We also analyzed the limiting data on patients without CKD as a control and confirmed the renal RI in non-CKD control group was significantly lower than that in patients with CKD (S2 Table).

**Table 1:** Baseline characteristics of the study subjects.

	All patients (n = 101)	Early stages CKD 1/2 (n = 25)	Mid stages CKD 3 (n = 29)	Later stages CKD 4/5 (n = 47)	P value
Age (years)	57.0 (42.0–68.8)	43.0 (31.5–57.5)	52.0 (43.0–61.5)	64.5 (50.8–71.8)	0.0003*
Male gender, n (%)	71 (68.9%)	17 (68.0%)	19 (65.5%)	35 (74.5%)	
Cause of CKD, n					
Glomerulonephritis	48 (47.5%)	16 (64.0%)	15 (51.8%)	17 (36.2%)	
Nephrosclerosis	25 (24.8%)	3 (12.0%)	8 (27.6%)	14 (29.8%)	
Diabetic nephropathy	12 (11.9%)	1 (4.0%)	3 (10.3%)	8 (17.0%)	
Others	16 (15.8%)	5 (20.0%)	3 (10.3%)	8 (17.0%)	
Current medication, n					
ARBs/ACEIs	54 (53.5%)	9 (36.0%)	13 (44.8%)	32 (68.1%)	
CCBs	53 (52.5%)	7 (28.0%)	12 (41.4%)	34 (72.3%)	
SBP (mmHg)	138 (124–150)	136 (123–143)	127 (115–141)	143 (130–158)	0.0008*
DBP (mmHg)	80 (72–89)	80 (72–89)	80 (68–88)	82 (74–90)	0.5371
Renal length (mm) Right	9.7 (8.9–10.2)	10.0 (9.6–10.3)	9.8 (8.9–10.4)	9.2 (8.6–10.0)	0.0082*
Left	9.7 (9.2–10.5)	10.5 (9.8–10.8)	9.8 (9.3–10.5)	9.3 (8.7–9.8)	< 0.0001*
Resistive Index (average)	0.66 (0.61–0.73)	0.62 (0.58–0.65)	0.63 (0.58–0.69)	0.73 (0.68–0.77)	< 0.0001*
Serum creatinine (μmol/L)	123.8 (81.3–260.8)	65.4 (52.2–78.7)	104.3 (1.05–1.38)	3.35 (2.22–4.72)	< 0.0001*
eGFR (mL/min/1.73m^2^)	39.7 (15.4–67.4)	84.1 (72.3–97.3)	49.9 (40.6–56.5)	14.5 (9.4–24.3)	< 0.0001*
Cystatin C (mg/L)	1.67 (0.97–3.40)	0.89 (0.77–0.94)	1.31 (1.08–1.66)	3.64 (2.36–4.01)	< 0.0001*
Hemoglobin (g/L)	130 (103–141)	139 (136–155)	139 (122–151)	102 (96–119)	< 0.0001*
Serum albumin (g/L)	40 (36–43)	42 (38–45)	42 (39–44)	37 (33–42)	0.0066*
Serum calcium (mmol/L)	2.22 (2.12–2.32)	2.32 (2.22–2.37)	2.27 (2.17–2.32)	2.15 (2.05–2.22)	< 0.0001*
Serum phosphate (mmol/L)	1.13 (1.03–1.36)	1.03 (0.87–1.19)	1.10 (1.00–1.26)	1.26 (1.10–1.55)	< 0.0001*
Uric acid (μmol/L)	422 (351–506)	351 (303–422)	375 (327–482)	464 (416–523)	< 0.0001*
Total-cholesterol (mmol/L)	4.86 (4.32–5.59)	4.81 (4.37–5.46)	5.53 (4.73–5.97)	4.65 (4.16–5.25)	0.0051*
LDL-cholesterol (mmol/L)	2.92 (2.40–3.59)	2.92 (2.51–3.67)	3.44 (2.87–3.88)	2.59 (2.17–3.10)	0.0011*
HDL-cholesterol (mmol/L)	1.34 (1.11–1.66)	1.50 (1.27–1.78)	1.40 (1.14–1.86)	1.27 (1.01–1.37)	0.0063*
HbA1c (NGSP) (%)	5.7 (5.5–6.1)	5.5 (5.5–6.0)	5.8 (5.5–5.9)	5.8 (5.6–6.1)	0.4754
FPG (mmol/L)	5.3 (4.9–6.0)	5.6 (5.1–6.6)	5.2 (4.8–5.9)	5.2 (4.9–6.0)	0.0759
Albuminuria (mg/day)	685 (252–1340)	347 (175–787)	443 (159–687)	1317 (884–2247)	< 0.0001*
Urinary β2MG (μg/L)	0.21 (0.09–2.43)	0.12 (0.07–0.20)	0.10 (0.07–0.40)	2.38 (0.37–10.32)	< 0.0001*
Intact PTH (ng/L)	56 (39–155)	39 (34–49)	46 (33–56)	155 (92–234)	< 0.0001*
BNP (ng/L)	23.3 (7.4–70.1)	8.2 (5.6–23.6)	12.8 (4.8–29.7)	59.5 (26.5–108.6)	< 0.0001*
baPWV (cm/sec, average)	1539 (1301–1890)	1350 (1217–1587)	1463 (1205–1743)	1758 (1475–2203)	< 0.0001*
Max IMT (mm, average)	0.83 (0.65–1.08)	0.76 (0.59–0.94)	0.86 (0.66–1.05)	0.86 (0.75–1.28)	0.1518
e’ (s)	6.3 (4.7–9.4)	9.7 (6.8–11.5)	6.3 (3.9–9.7)	5.6 (4.6–7.2)	0.0010*
EF (%)	67 (62–71)	67 (65–71)	68 (62–72)	67 (60–70)	0.2973

ACEI, angiotensin converting enzyme inhibitor; ARB, angiotensin receptor blocker; baPWV, brachial-ankle pulse wave velocity; DBP, diastolic blood pressure; eGFR, estimated glomerular filtration rate; FPG, fasting plasma glucose; HDL, high density lipoprotein; IMT, intima-media thickness; LDL, low density lipoprotein; NGSP, national glycohemoglobin standardization program; SBP, systolic blood pressure.

*Statistically significant.

### The correlations between the resistive index and the clinical and laboratory Indexes, and markers of systemic atherosclerosis

**Fig 1:**
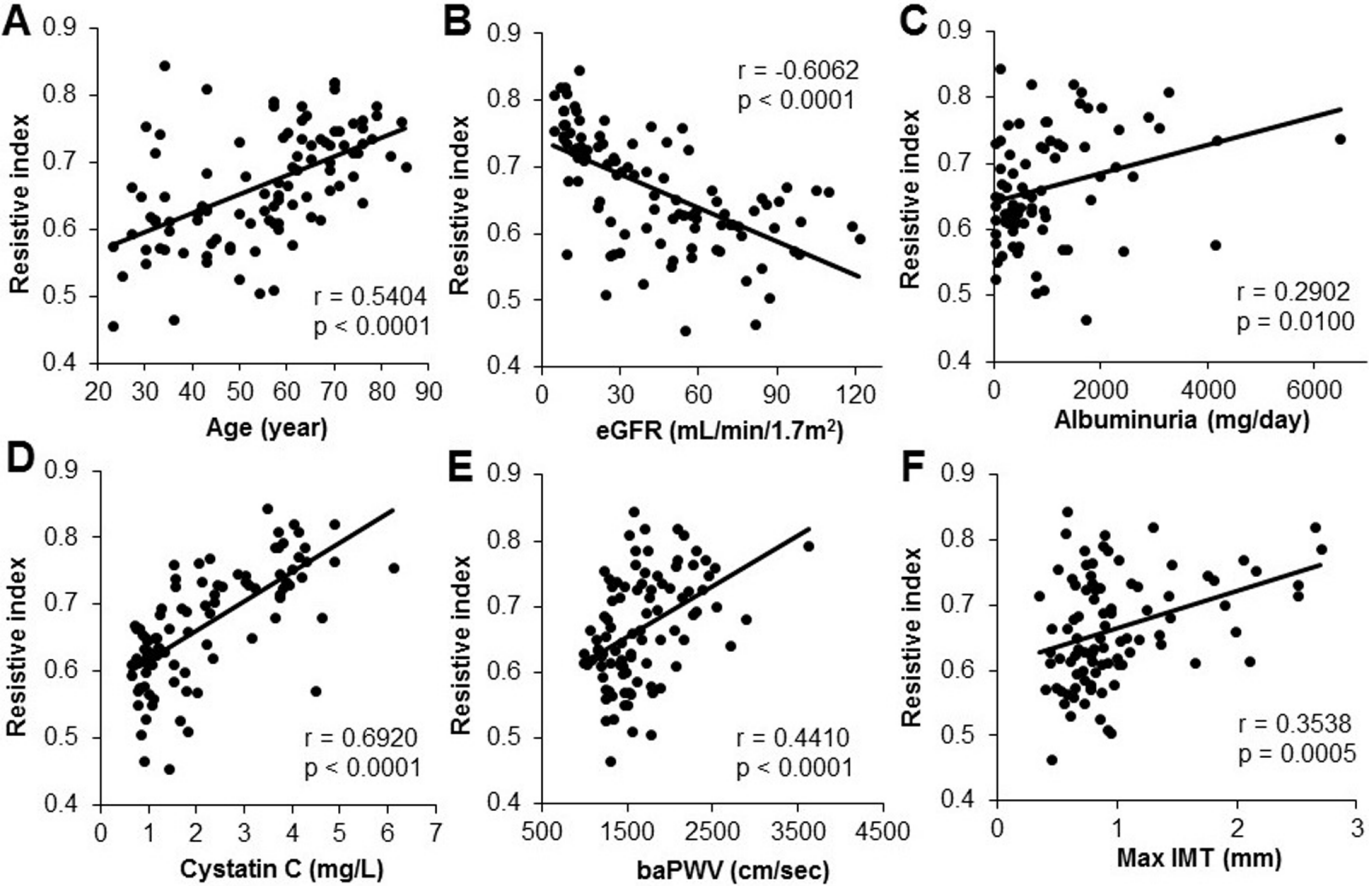
The correlation between the resistive index (RI) and various parameters. The relationships between the RI and patient age (years) (A), estimated glomerular filtration rate (eGFR) (mL/min/1.73 m2) (B), albuminuria (mg/day) (C), cystatin C (mg/L) (D) and markers of systemic atherosclerosis, including the ankle-brachial pulse wave velocity (baPWV) (E) and maximum intima-media thickness (IMT) (F), are shown. The RI was positively correlated with age, albuminuria, and cystatin C, and inversely correlated with eGFR (A-D). Regarding the markers of systemic atherosclerosis, baPWV and maximum IMT were positively correlated with RI (E, F).

A univariate analysis revealed a significant negative correlation between the RI, as detected by Doppler echography, and the eGFR (*P* < 0.0001, r = - 0.6062; Fig 1B); similar findings have been reported in CKD patients [30, 31] Significant correlations were also observed between the RI and age (P < 0.0001, r = 0.5404; Fig 1A), albuminuria (P = 0.0100, r = 0.2902; Fig 1C), cystatin C (P < 0.0001, r = 0.6920; Fig 1D), baPWV (P < 0.0001, r = 0.4410; Fig 1E), and maximum IMT (P = 0.0005, r = 0.3538; Fig 1F). According to the cause of CKD, significant correlations were also observed between the RI, eGFR, and serum cystatin C in glomerulonephritis or nephrosclerosis group (S3 Table).

### The multivariate analysis of the determinants of the RI

**Fig 2:**
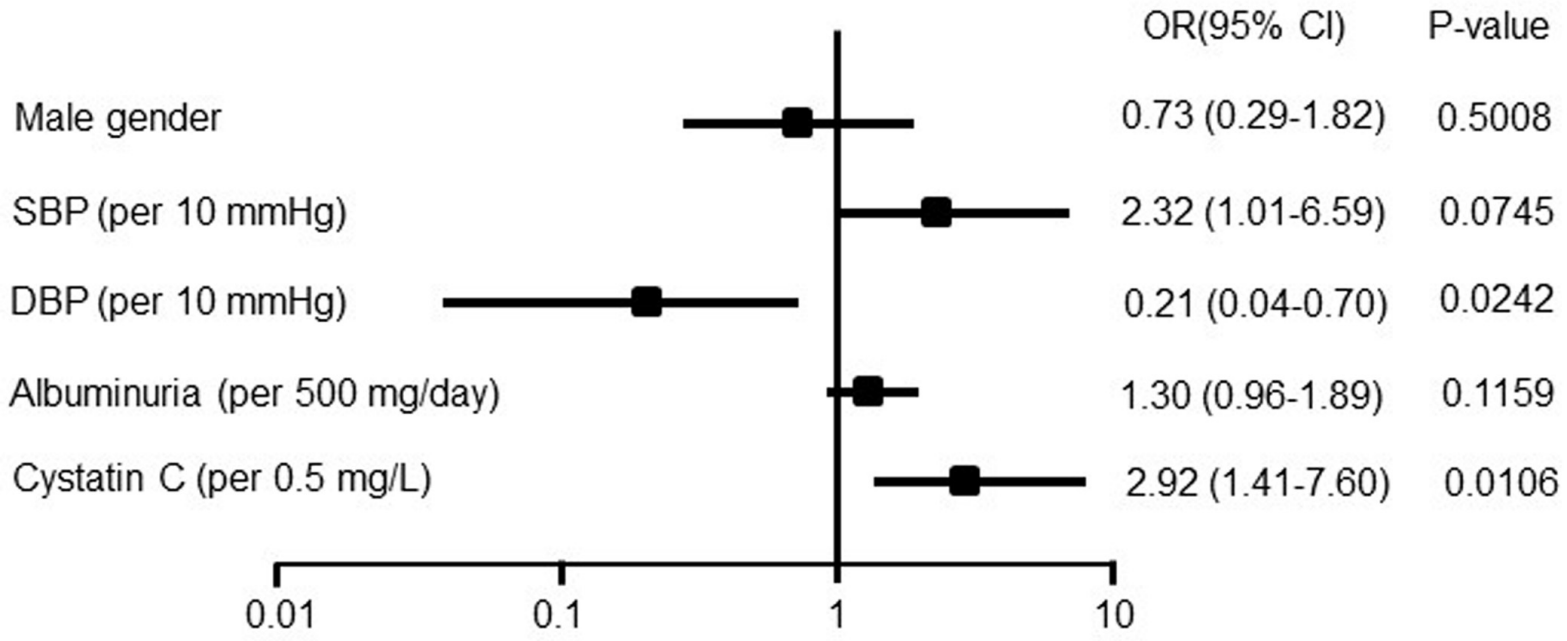
The multivariate odds ratios for the resistive index (RI: 0.66) among patients with CKD. The values are displayed as the odds ratio (OR) (solid boxes) with 95% confidence intervals (CIs) (horizontal limit lines). For continuous variables, the unit of change is given in parentheses. Adjusted for age and eGFR. SBP, systolic blood pressure; DBP, diastolic blood pressure; eGFR, estimated glomerular filtration rate.

Table 2 shows separate multivariate analysis models for the RI. After adjustment for age, gender, blood pressure (systolic and diastolic), and eGFR, the factors that were expected to influence the RI were albuminuria in the CKD model; baPWV and max IMT in the CVD model; and cystatin C in the biomarker model. The RI was significantly associated with the max IMT (P = 0.0143), e’ (P < 0.0001), cystatin C (P < 0.0001), and serum phosphate (P = 0.0093) values. Next, in the multivariate logistic regression model, cystatin C was significantly associated with higher odds of having an RI value of > 0.66 after adjustment with gender, blood pressure, and albuminuria (Fig 2). The odds ratios (ORs) for age (per 10-year increase) and cystatin C (per 0.5 mg/L increase) were 2.46 (95% CI: 1.36 to 5.16) and 2.92 (95% CI: 1.47 to 7.60), respectively (Fig 2). However, the odds ratios for the baPWV and the mean IMT were not significant (S4 and S5 Table).

**Table 2:** The multiple regression analysis of the predictors of the resistive index.

	independent variables	β	p	model r^2^
CKD model	Uric acid	-0.005919	0.2585	0.5206
	Albuminuria	-0.000001	0.2980	
CVD model	baPWV	-0.00001	0.5571	0.6736
	max IMT	0.031885	0.0143	
	e’	0.013688	< 0.0001	
Biomarker model	BNP	-0.00003	0.4898	0.6134
	Cystatin C	0.036144	< 0.0001	
MBD model	intact PTH	-0.00005	0.5056	0.6215
	Serum calcium	-0.015924	0.2372	
	Serum phosphate	0.008771	0.0093	

Adjusted for age, gender, blood pressure (systolic and diastolic), and eGFR.

baPWV, brachial-ankle pulse wave velocity; CKD, chronic kidney disease; CVD, cardio vascular disease; eGFR, estimated glomerular filtration rate; IMT, intima-media thickness; MBD, mineral and bone disorder; PTH, parathyroid hormone.

### The cystatin C level significantly increased in CKD patients with an RI of ≥ 0.66

**Fig 3:**
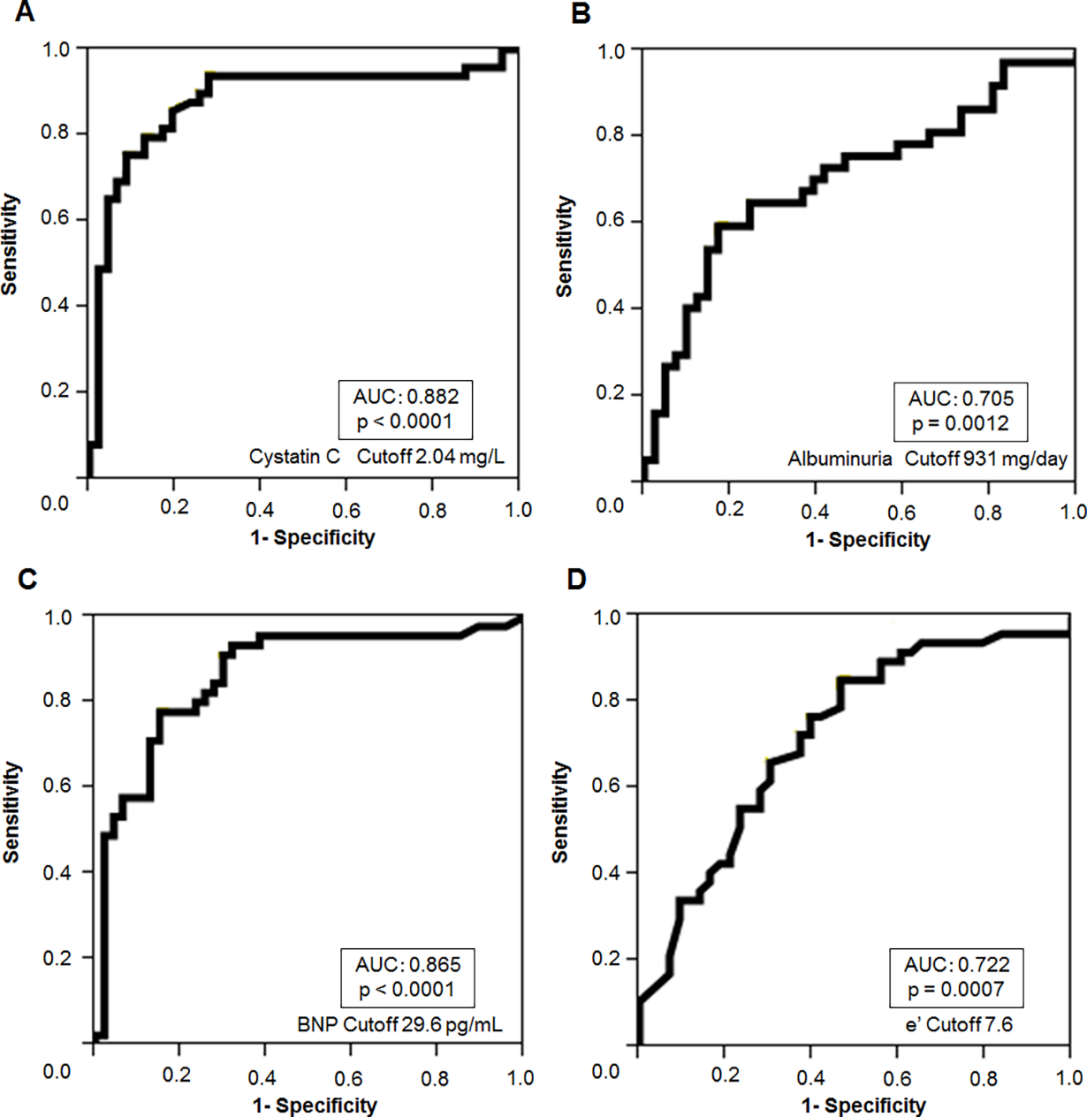
**The ROC curves comparing the sensitivity and specificity of cystatin C (A), albuminuria (B), BNP (C) and e’ (D) for predicting a resistive index (RI) 0.66.** The AUC values for the ROC curve when cystatin C, albuminuria, BNP and e’ were used to detect an RI of 0.66 were 0.882 (p < 0.0001), 0.705 (p = 0.0012), 0.865 (p < 0.0001) and 0.722 (p = 0.0007), respectively.

Fig 3 shows the ROC curves comparing the sensitivity and specificity of cystatin C (Fig 3A), albuminuria (Fig 3B), BNP (Fig 3C) and e’ (Fig 3D) for predicting an RI of 0.66. The AUC values of the ROC curve, when cystatin C, albuminuria, BNP, and e’ were used to detect an RI of ≥ 0.66, were 0.882 (P < 0.0001), 0.705 (P = 0.0012), 0.865 (P < 0.0001), and 0.722 (P = 0.0007), respectively. Thus, the AUC for cystatin C was the greatest in this model. For comparison, S1 Fig shows the ROC curves comparing the sensitivity and specificity of cystatin C for predicting a max IMT of 1.11 and a baPWV of 1400. The AUC values for the ROC curve, when cystatin C was used to detect a max IMT of ≥1.1 and a baPWV of ≥ 1400 were lower than those for an RI of ≥ 0.66.

## Discussion

In the present study, we measured the renal RI, markers of vascular dysfunction, including the baPWV and max IMT, and the cardiac function. Furthermore, we determined the relationships between the serum cystatin C level and markers of vascular dysfunction, including the renal RI, baPWV and max IMT, and the cardiac function in CKD patients. The serum cystatin C level is significantly correlated with the renal RI but not with other signs of vascular dysfunction, such as the baPWV or maximum IMT in multivariate models. Since no reports have described the relationship between serum cystatin C and renal RI in CKD patients, we believe this is the first study to show that serum cystatin C is an independent biomarker associated with the renal RI in patients with CKD.

Consistent with previous studies [15, 16, 32, 33], we found that the renal RI was correlated with age, eGFR, SBP, albuminuria, baPWV, and max IMT in CKD patients. Recently, numerous studies have reported that the RI may be a significant predictor of cardiovascular and renal outcomes [14, 18]. These results suggest that an increase in the renal RI of the intrarenal vasculature reflects a generalized increase in arteriosclerosis and a widening of the pulse pressure.

Furthermore, the renal RI was correlated with the serum cystatin C level in this study. Although the serum cystatin C level was correlated with age, eGFR, albuminuria, and baPWV, as previously reported [34, 35], a multivariate analysis revealed that the serum cystatin C level was also a significant predictor of the renal RI in a biomarker model after adjustment for age, gender, blood pressure, and the eGFR. In addition, the multivariate odds ratio of the serum cystatin C level for a renal RI of 0.66 (the median value that predicts worse renal outcomes [18] and that is higher than a renal RI of 0.60 in non-CKD controls), was significant. However, the odds ratios of the serum cystatin C level for other systemic atherosclerosis indexes, including the baPWV (ORs = 1.13, P = 0.6317) and the max IMT (ORs = 1.09, P = 0.7657), were not significant.

A recent study reported that the number of patients needing renal replacement therapy is increasing [36]. End-stage kidney disease is a leading cause of morbidity and mortality worldwide; thus, it is important to detect a biomarker of both the renal outcome and mortality. In addition, cardiovascular disease is frequently associated with CKD, which is important, since individuals with CKD are more likely to die of cardiovascular disease than to develop kidney failure. Indeed, the term “cardiorenal syndrome” has been increasingly used, and a new classification was proposed because a large proportion of patients admitted to hospital have various degrees of heart and kidney dysfunction [37]. As previously reported [13, 18, 19], the renal RI predicts the renal prognosis, cardiovascular events, and death in CKD patients. Moreover, the cystatin C concentration predicts all-cause and CVD mortality in patients with CKD [5, 38], and cystatin C might be a biomarker of cardiac dysfunction and hypertrophy [6, 22]. Thus, once a patient is diagnosed with CKD, the measurement of both the RI and the serum cystatin C level appears to be useful for predicting the renal outcome, cardiovascular damage, and the risk of cardiovascular events. Our findings provide valuable insight into the relationship between the cystatin C level and a renal RI of 0.66 (the median level at which worse renal and cardiovascular outcomes are predicted [18]). We reported that the cystatin C level could predict this RI value in patients with a moderate degree of accuracy (AUC = 0.882, Cut off cystatin C value: 2.04mg/L). Incidentally, the AUC values of albuminuria and BNP for predicting this value (RI 0.66) were lower than the AUC of cystatin C.

Our study is associated with some limitations and strengths that should be kept in mind when interpreting the results. First, the cross-sectional nature of our observations precluded making any inferences with regard to cause and effect in the relationship between the serum cystatin C level and the renal RI in CKD patients. Secondly, we could not adjust the drugs that may affect the serum cystatin C concentration (*i*.*e*., corticosteroids). However, this weakness was—in part—offset by the timing of the measurement because we examined the serum cystatin C level earlier than initiation of immunosuppressive therapy or in the predialysis stage. Thus, most of the participants were not using these drugs. Thirdly, the number of subjects in this study was relatively small. Fourthly, we did not evaluate the intrarenal venous flow patterns with renal Doppler ultrasonography, which was recently suggested to be associated with cardiovascular mortality in patients with heart failure [39].

In conclusion, the serum cystatin C level is independently associated with signs of vascular dysfunction, such as the renal RI in patients with CKD. Recent studies indicate the prognostic significance of the renal RI after renal artery angioplasty and stenting for atherosclerotic renal artery stenosis [40] or for flash pulmonary edema [41]. Further study to elucidate whether or not serum cystatin C can replace the renal RI in such clinical settings would be of interest.

## Supporting information

S1 Table Baseline characteristics of the study subjects according to the cause of CKD. (DOCX)

S2 Table Baseline characteristics of the control subjects and all patients. (DOCX)

S3 Table The correlation between the RI and various parameters according to the cause of CKD. (DOCX)

S4 Table The multivariate odds ratios (95% CI) for baPWV ≥ 1400. (DOCX)

S5 Table The multivariate odds ratios (95% CI) for Max IMT ≥ 1.1. (DOCX)

S1 Fig **The ROC curves comparing the sensitivity and specificity of cystatin C for predicting a maximum intima-media thickness (IMT) of 1.1 (A) and an ankle-brachial pulse wave velocity (baPWV) of 1400 (B).** The AUC values for the ROC curves when cystatin C was used to detect a maximum IMT of 1.1 and a baPWV of 1400, were 0.675 (p = 0.0434) and 0.753 (p = 0.0012), respectively.
(TIF)

## References

[1] Shlipak MG, Sarnak MJ, Katz R, Fried LF, Seliger SL, Newman AB, et al Cystatin C and the risk of death and cardiovascular events among elderly persons. N Engl J Med. 2005;352(20):2049–60. doi: 10.1056/NEJMoa043161 . 15901858 10.1056/NEJMoa043161

[2] Shlipak MG, Praught ML, Sarnak MJ. Update on cystatin C: new insights into the importance of mild kidney dysfunction. Curr Opin Nephrol Hypertens. 2006;15(3):270–5. doi: 10.1097/01.mnh.0000222694.07336.92 . 16609294 10.1097/01.mnh.0000222694.07336.92

[3] Coll E, Botey A, Alvarez L, Poch E, Quinto L, Saurina A, et al Serum cystatin C as a new marker for noninvasive estimation of glomerular filtration rate and as a marker for early renal impairment. Am J Kidney Dis. 2000;36(1):29–34. doi: 10.1053/ajkd.2000.8237 . 10873868 10.1053/ajkd.2000.8237

[4] Dharnidharka VR, Kwon C, Stevens G. Serum cystatin C is superior to serum creatinine as a marker of kidney function: a meta-analysis. Am J Kidney Dis. 2002;40(2):221–6. doi: 10.1053/ajkd.2002.34487 . 12148093 10.1053/ajkd.2002.34487

[5] Ix JH, Shlipak MG, Chertow GM, Whooley MA. Association of cystatin C with mortality, cardiovascular events, and incident heart failure among persons with coronary heart disease: data from the Heart and Soul Study. Circulation. 2007;115(2):173–9. doi: 10.1161/CIRCULATIONAHA.106.644286 ; PubMed Central PMCID: PMC2771187. 17190862 10.1161/CIRCULATIONAHA.106.644286 PMC2771187

[6] Prats M, Font R, Bardaji A, Gutierrez C, Lalana M, Vilanova A, et al Cystatin C and cardiac hypertrophy in primary hypertension. Blood Press. 2010;19(1):20–5. doi: 10.3109/08037050903416386 . 20113216 10.3109/08037050903416386

[7] Izumi M, Sugiura T, Nakamura H, Nagatoya K, Imai E, Hori M. Differential diagnosis of prerenal azotemia from acute tubular necrosis and prediction of recovery by Doppler ultrasound. Am J Kidney Dis. 2000;35(4):713–9. . 10739794 10.1016/s0272-6386(00)70020-5

[8] Boddi M, Cecioni I, Poggesi L, Fiorentino F, Olianti K, Berardino S, et al Renal resistive index early detects chronic tubulointerstitial nephropathy in normo- and hypertensive patients. Am J Nephrol. 2006;26(1):16–21. doi: 10.1159/000090786 . 16401882 10.1159/000090786

[9] Ikee R, Kobayashi S, Hemmi N, Imakiire T, Kikuchi Y, Moriya H, et al Correlation between the resistive index by Doppler ultrasound and kidney function and histology. Am J Kidney Dis. 2005;46(4):603–9. doi: 10.1053/j.ajkd.2005.06.006 . 16183414 10.1053/j.ajkd.2005.06.006

[10] Sugiura T, Nakamori A, Wada A, Fukuhara Y. Evaluation of tubulointerstitial injury by Doppler ultrasonography in glomerular diseases. Clin Nephrol. 2004;61(2):119–26. . 14989631 10.5414/cnp61119

[11] Planiol T, Pourcelot L, Itti R. [Radioisotopes, ultrasonics and thermography in the diagnosis of cerebral circulatory disorders]. Rev Electroencephalogr Neurophysiol Clin. 1974;4(2):221–36. . 4608395 10.1016/s0370-4475(74)80006-7

[12] Petersen LJ, Petersen JR, Talleruphuus U, Ladefoged SD, Mehlsen J, Jensen HA. The pulsatility index and the resistive index in renal arteries. Associations with long-term progression in chronic renal failure. Nephrol Dial Transplant. 1997;12(7):1376–80. . 9249772 10.1093/ndt/12.7.1376

[13] Sugiura T, Wada A. Resistive index predicts renal prognosis in chronic kidney disease. Nephrol Dial Transplant. 2009;24(9):2780–5. doi: 10.1093/ndt/gfp121 . 19318356 10.1093/ndt/gfp121

[14] Sugiura T, Wada A. Resistive index predicts renal prognosis in chronic kidney disease: results of a 4-year follow-up. Clin Exp Nephrol. 2011;15(1):114–20. doi: 10.1007/s10157-010-0371-3 . 21069409 10.1007/s10157-010-0371-3

[15] Ohta Y, Fujii K, Arima H, Matsumura K, Tsuchihashi T, Tokumoto M, et al Increased renal resistive index in atherosclerosis and diabetic nephropathy assessed by Doppler sonography. J Hypertens. 2005;23(10):1905–11. . 16148615 10.1097/01.hjh.0000181323.44162.01

[16] Heine GH, Reichart B, Ulrich C, Kohler H, Girndt M. Do ultrasound renal resistance indices reflect systemic rather than renal vascular damage in chronic kidney disease? Nephrol Dial Transplant. 2007;22(1):163–70. doi: 10.1093/ndt/gfl484 . 16936334 10.1093/ndt/gfl484

[17] Kawai T, Kamide K, Onishi M, Yamamoto-Hanasaki H, Baba Y, Hongyo K, et al Usefulness of the resistive index in renal Doppler ultrasonography as an indicator of vascular damage in patients with risks of atherosclerosis. Nephrol Dial Transplant. 2011;26(10):3256–62. doi: 10.1093/ndt/gfr054 . 21372256 10.1093/ndt/gfr054

[18] Doi Y, Iwashima Y, Yoshihara F, Kamide K, Hayashi S, Kubota Y, et al Renal resistive index and cardiovascular and renal outcomes in essential hypertension. Hypertension. 2012;60(3):770–7. doi: 10.1161/HYPERTENSIONAHA.112.196717 . 22824987 10.1161/HYPERTENSIONAHA.112.196717

[19] Toledo C, Thomas G, Schold JD, Arrigain S, Gornik HL, Nally JV, et al Renal Resistive Index and Mortality in Chronic Kidney Disease. Hypertension. 2015;66(2):382–8. doi: 10.1161/HYPERTENSIONAHA.115.05536 ; PubMed Central PMCID: PMC4498966. 26077569 10.1161/HYPERTENSIONAHA.115.05536 PMC4498966

[20] Kidney Disease Outcomes Quality I. K/DOQI clinical practice guidelines on hypertension and antihypertensive agents in chronic kidney disease. Am J Kidney Dis. 2004;43(5 Suppl 1):S1–290. . 15114537

[21] Matsuo S, Imai E, Horio M, Yasuda Y, Tomita K, Nitta K, et al Revised equations for estimated GFR from serum creatinine in Japan. Am J Kidney Dis. 2009;53(6):982–92. doi: 10.1053/j.ajkd.2008.12.034 . 19339088 10.1053/j.ajkd.2008.12.034

[22] Nosaka K, Nakamura K, Kusano K, Toh N, Tada T, Miyoshi T, et al Serum cystatin C as a biomarker of cardiac diastolic dysfunction in patients with cardiac disease and preserved ejection fraction. Congest Heart Fail. 2013;19(4):E35–9. doi: 10.1111/chf.12039 . 23910705 10.1111/chf.12039

[23] Nakamura A, Shikata K, Hiramatsu M, Nakatou T, Kitamura T, Wada J, et al Serum interleukin-18 levels are associated with nephropathy and atherosclerosis in Japanese patients with type 2 diabetes. Diabetes Care. 2005;28(12):2890–5. . 16306550 10.2337/diacare.28.12.2890

[24] Kitagawa M, Sugiyama H, Morinaga H, Inoue T, Takiue K, Ogawa A, et al A Decreased Level of Serum Soluble Klotho Is an Independent Biomarker Associated with Arterial Stiffness in Patients with Chronic Kidney Disease. PLoS One. 2013;8(2):e56695 doi: 10.1371/journal.pone.0056695 23431388 10.1371/journal.pone.0056695 PMC3576368

[25] Yamashina A, Tomiyama H, Arai T, Hirose K, Koji Y, Hirayama Y, et al Brachial-ankle pulse wave velocity as a marker of atherosclerotic vascular damage and cardiovascular risk. Hypertens Res. 2003;26(8):615–22. . 14567500 10.1291/hypres.26.615

[26] Handa N, Matsumoto M, Maeda H, Hougaku H, Ogawa S, Fukunaga R, et al Ultrasonic evaluation of early carotid atherosclerosis. Stroke. 1990;21(11):1567–72. . 2237950 10.1161/01.str.21.11.1567

[27] Kitagawa M, Sugiyama H, Morinaga H, Inoue T, Takiue K, Kikumoto Y, et al Serum high-sensitivity cardiac troponin T is a significant biomarker of left-ventricular diastolic dysfunction in subjects with non-diabetic chronic kidney disease. Nephron extra. 2011;1(1):166–77. doi: 10.1159/000333801 ; PubMed Central PMCID: PMC3290834. 22470390 10.1159/000333801 PMC3290834

[28] Schiller NB, Shah PM, Crawford M, DeMaria A, Devereux R, Feigenbaum H, et al Recommendations for quantitation of the left ventricle by two-dimensional echocardiography. American Society of Echocardiography Committee on Standards, Subcommittee on Quantitation of Two-Dimensional Echocardiograms. J Am Soc Echocardiogr. 1989;2(5):358–67. . 2698218 10.1016/s0894-7317(89)80014-8

[29] Nagueh SF, Middleton KJ, Kopelen HA, Zoghbi WA, Quinones MA. Doppler tissue imaging: a noninvasive technique for evaluation of left ventricular relaxation and estimation of filling pressures. J Am Coll Cardiol. 1997;30(6):1527–33. . 9362412 10.1016/s0735-1097(97)00344-6

[30] Parolini C, Noce A, Staffolani E, Giarrizzo GF, Costanzi S, Splendiani G. Renal resistive index and long-term outcome in chronic nephropathies. Radiology. 2009;252(3):888–96. doi: 10.1148/radiol.2523080351 . 19528356 10.1148/radiol.2523080351

[31] Bige N, Levy PP, Callard P, Faintuch JM, Chigot V, Jousselin V, et al Renal arterial resistive index is associated with severe histological changes and poor renal outcome during chronic kidney disease. BMC Nephrol. 2012;13:139 doi: 10.1186/1471-2369-13-139 ; PubMed Central PMCID: PMC3531254. 23098365 10.1186/1471-2369-13-139 PMC3531254

[32] Kimura N, Kimura H, Takahashi N, Hamada T, Maegawa H, Mori M, et al Renal resistive index correlates with peritubular capillary loss and arteriosclerosis in biopsy tissues from patients with chronic kidney disease. Clin Exp Nephrol. 2015 doi: 10.1007/s10157-015-1116-0 . 26081566 10.1007/s10157-015-1116-0

[33] Hamano K, Nitta A, Ohtake T, Kobayashi S. Associations of renal vascular resistance with albuminuria and other macroangiopathy in type 2 diabetic patients. Diabetes Care. 2008;31(9):1853–7. doi: 10.2337/dc08-0168 ; PubMed Central PMCID: PMC2518358. 18566339 10.2337/dc08-0168 PMC2518358

[34] Odden MC, Tager IB, Gansevoort RT, Bakker SJ, Katz R, Fried LF, et al Age and cystatin C in healthy adults: a collaborative study. Nephrol Dial Transplant. 2010;25(2):463–9. doi: 10.1093/ndt/gfp474 ; PubMed Central PMCID: PMC2904248. 19749145 10.1093/ndt/gfp474 PMC2904248

[35] Yamashita H, Nishino T, Obata Y, Nakazato M, Inoue K, Furusu A, et al Association between cystatin C and arteriosclerosis in the absence of chronic kidney disease. J Atheroscler Thromb. 2013;20(6):548–56. . 23574755 10.5551/jat.13193

[36] Liyanage T, Ninomiya T, Jha V, Neal B, Patrice HM, Okpechi I, et al Worldwide access to treatment for end-stage kidney disease: a systematic review. Lancet. 2015;385(9981):1975–82. doi: 10.1016/S0140-6736(14)61601-9 . 25777665 10.1016/S0140-6736(14)61601-9

[37] Ronco C, Haapio M, House AA, Anavekar N, Bellomo R. Cardiorenal syndrome. J Am Coll Cardiol. 2008;52(19):1527–39. doi: 10.1016/j.jacc.2008.07.051 . 19007588 10.1016/j.jacc.2008.07.051

[38] Menon V, Shlipak MG, Wang X, Coresh J, Greene T, Stevens L, et al Cystatin C as a risk factor for outcomes in chronic kidney disease. Ann Intern Med. 2007;147(1):19–27. 17606957 10.7326/0003-4819-147-1-200707030-00004

[39] Iida N, Seo Y, Sai S, Machino-Ohtsuka T, Yamamoto M, Ishizu T, et al Clinical Implications of Intrarenal Hemodynamic Evaluation by Doppler Ultrasonography in Heart Failure. JACC Heart Fail. 2016;4(8):674–82. doi: 10.1016/j.jchf.2016.03.016 . 27179835 10.1016/j.jchf.2016.03.016

[40] Yuksel UC, Anabtawi AG, Cam A, Poddar K, Agarwal S, Goel S, Kim E, Bajzer C, Gornik HL, Shishehbor MH, Tuzcu EM, Kapadia SR. Predictive value of renal resistive index in percutaneous renal interventions for atherosclerotic renal artery stenosis. J Invasive Cardiol. 2012;24(10):504–9. . 23043033

[41] Delsart P, Meurice J, Midulla M, Bauters C, Haulon S, Mounier-Vehier C. Prognostic Significance of the Renal Resistive Index After Renal Artery Revascularization in the Context of Flash Pulmonary Edema. J Endovasc Ther. 2015 10;22(5):801–5. doi: 10.1177/1526602815599964 . 26250746 10.1177/1526602815599964

