# Supplementary material for: Serum cystatin C is an independent biomarker associated with the renal resistive index in patients with chronic kidney disease
Source: PLoS One. 2018 Mar 7;13(3):e0193695. doi: 10.1371/journal.pone.0193695 (PMC5841772; doi:10.1371/journal.pone.0193695)
Supplement: S1 Table — (DOCX) [file pone.0193695.s001.docx]

**S1 Table. Baseline characteristics of the study subjects according to the cause of CKD.**

|  | Glomerulonephritis  (n =48) | Nephrosclerosis  (n = 25) | Diabetic nephropathy  (n = 12) | P value |
| --- | --- | --- | --- | --- |
| Age (years) | 48.0 (34.0-61.0) | 64.0 (57.5-73.5) | 67.0 (57.5-74.0) | < 0.0001* |
| Male gender, n (%) | 32 (66.7%) | 20 (80.0%) | 8 (66.7%) |  |
| Current medication, n |  |  |  |  |
| ARBs/ACEIs | 18 (37.5%) | 18 (72.0%) | 10 (83.3%) |  |
| CCBs | 15 (31.3%) | 18 (72.0%) | 11 (91.7%) |  |
| SBP (mmHg) | 134 (122-142) | 138 (119-153) | 143 (135-156) | 0.1721 |
| DBP (mmHg) | 80 (74-89) | 79 (65-85) | 84 (78-89) | 0.1673 |
| Renal length (mm) Right | 9.8 (9.3-10.3) | 9.3 (8.5-9.8) | 10.1 (8.8-11.0) | 0.0412* |
| Left | 9.9 (9.3-10.5) | 9.3 (8.8-9.7) | 9.8 (9.5-11.0) | 0.0162* |
| Resistive Index (average) | 0.63 (0.58-0.68) | 0.71 (0.65-0.75) | 0.75 (0.70-0.79) | < 0.0001* |
| Serum creatinine (μmol/L) | 93.7 (71.6-186.5) | 165.3 (111.4-122.0) | 203.3 (100.8-362.4) | 0.0062* |
| eGFR (mL/min/1.73m^2^) | 56.5 (23.7-75.1) | 27.6 (13.3-42.6) | 24.0 (12.5-45.3) | 0.0035* |
| Cystatin C (mg/L) | 1.08 (0.92-2.24) | 2.36 (1.21-3.63) | 2.32 (1.52-3.78) | 0.0021* |
| Hemoglobin (g/L) | 137 (115-144) | 119 (100-139) | 103 (96-112) | 0.0042* |
| Serum albumin (g/L) | 40 (36-43) | 43 (38-45) | 36 (32-38) | 0.0057* |
| Serum calcium (mmol/L) | 2.22 (2.15-2.32) | 2.27 (2.16-2.33) | 2.10 (1.91-2.18) | 0.0042* |
| Serum phosphate (mmol/L) | 1.13 (1.00-1.23) | 1.13 (1.03-1.36) | 1.32 (1.05-1.70) | 0.2546 |
| Uric acid (μmol/L) | 393 (333-489) | 476 (381-515) | 404 (320-454) | 0.0861 |
| Total-cholesterol (mmol/L) | 5.04 (4.48-5.78) | 4.65 (4.19-5.18) | 4.90 (4.37-5.49) | 0.1098 |
| LDL-cholesterol (mmol/L) | 3.04 (2.51-3.83) | 2.74 (2.20-3.18) | 2.62 (2.31-2.97) | 0.0806 |
| HDL-cholesterol (mmol/L) | 1.37 (1.14-1.71) | 1.24 (1.02-1.66) | 1.33 (1.11-1.51) | 0.5365 |
| HbA1c (NGSP) (%) | 5.6 (5.4-5.9) | 5.7 (5.5-5.8) | 6.6 (6.3-7.5) | 0.0002* |
| FPG (mmol/L) | 5.3 (4.9-5.7) | 5.4 (5.0-5.9) | 6.7 (5.9-9.0) | 0.0003* |
| Albuminuria (mg/day) | 693 (348-1543) | 286 (107-968) | 1629 (601-3078) | 0.0079* |
| Urinary β2MG (μg/L) | 0.18 (0.08-0.58) | 0.22 (0.08-3.48) | 1.76 (0.34-14.16) | 0.0251* |
| Intact PTH (ng/L) | 45 (35-92) | 119 (51-167) | 146 (56-242) | 0.0083* |
| BNP (ng/L) | 13.9 (6.0-35.4) | 66.2 (18.8-149.0) | 44.7(15.2-196.3) | 0.0029* |
| baPWV (cm/sec, average) | 1437 (1257-1744) | 1719 (1493-2057) | 2070 (1597-2347) | 0.0029* |
| Max IMT (mm, average) | 0.78 (0.59-1.00) | 0.96 (0.83-1.30) | 0.95 (0.89-1.86) | 0.0014* |
| e’ (s) | 7.5 (5.4-10.8) | 5.5 (4.2-7.2) | 5.1 (3.8-6.0) | 0.0020* |
| EF (%) | 66 (61-72) | 68 (62-72) | 67 (62-69) | 0.8076 |

ACEI, angiotensin converting enzyme inhibitor; ARB, angiotensin receptor blocker; baPWV, brachial-ankle pulse wave velocity; DBP, diastolic blood pressure; e’, early peak diastolic annular velocity of mitral valves; EF, ejection fraction; eGFR, estimated glomerular filtration rate; FPG, free plasma glucose; HDL, high density lipoprotein; IMT, intima-media thickness; LDL, low density lipoprotein; NGSP, national glycohemoglobin standardization program; SBP, systolic blood pressure.
