# Supplementary material for: Serum cystatin C is an independent biomarker associated with the renal resistive index in patients with chronic kidney disease
Source: PLoS One. 2018 Mar 7;13(3):e0193695. doi: 10.1371/journal.pone.0193695 (PMC5841772; doi:10.1371/journal.pone.0193695)
Supplement: S2 Table — (DOCX) [file pone.0193695.s002.docx]

**S2 Table. Baseline characteristics of the control subjects and all patients.**

|  | Control  (n = 14) | All patients  (n = 101) | P value |
| --- | --- | --- | --- |
| Age (years) | 41.5 (22.0-60.3) | 57.0 (42.0-68.8) | 0.0216* |
| Male gender, n (%) | 10 (71.4%) | 71 (68.9%) |  |
| SBP (mmHg) | 123 (109-148) | 138 (124-150) | 0.1150 |
| DBP (mmHg) | 74 (59-87) | 80 (72-89) | 0.0484* |
| Renal length (mm) Right | 10.9 (10.2-11.1) | 9.7 (8.9-10.2) | 0.0001* |
| Left | 11.1 (10.1-11.5) | 9.7 (9.2-10.5) | 0.0002* |
| Resistive Index (average) | 0.60 (0.58-0.63) | 0.66 (0.61-0.73) | 0.0091* |
| Serum creatinine (μmol/L) | 72.0 (55.3-79.8) | 123.8 (81.3-260.8) | < 0.0001* |
| eGFR (mL/min/1.73m^2^) | 87.6 (71.9-112.5) | 39.7 (15.4-67.4) | < 0.0001* |
| HbA1c (NGSP) (%) | 5.6 (5.4-6.0) | 5.7 (5.5-6.1) | 0.4936 |

Control, non-CKD patients; DBP, diastolic blood pressure; eGFR, estimated glomerular filtration rate; NGSP, national glycohemoglobin standardization program; SBP, systolic blood pressure.
