# Supplementary material for: Serum cystatin C is an independent biomarker associated with the renal resistive index in patients with chronic kidney disease
Source: PLoS One. 2018 Mar 7;13(3):e0193695. doi: 10.1371/journal.pone.0193695 (PMC5841772; doi:10.1371/journal.pone.0193695)
Supplement: S3 Table — (DOCX) [file pone.0193695.s003.docx]

**S3 Table. The correlation between the RI and various parameters according to the cause of CKD.**

| Parameter | Glomerulonephritis (n = 48) | | Nephrosclerosis (n =25) | | Diabetic nephropathy (n = 12) | |
| --- | --- | --- | --- | --- | --- | --- |
|  | r | P-value | r | P-value | r | P-value |
| Age | 0.4989 | 0.0004* | 0.2513 | 0.2255 | 0.0117 | 0.9712 |
| eGFR (mL/min/1.73m^2^) | 0.4913 | 0.0004* | 0.7013 | < 0.0001* | 0.3375 | 0.2833 |
| Albuminuria (mg/day) | 0.2521 | 0.1073 | 0.4672 | 0.0506 | 0.2224 | 0.5652 |
| Cystatin C (mg/L) | 0.6086 | < 0.0001* | 0.7560 | < 0.0001* | 0.5467 | 0.0659 |
| baPWV (cm/sec) | 0.3085 | 0.0392* | 0.2576 | 0.2470 | 0.3844 | 0.2173 |
| Max IMT (mm) | 0.3878 | 0.0071* | 0.1917 | 0.3928 | 0.0387 | 0.9155 |

baPWV, brachial-ankle pulse wave velocity; eGFR, estimated glomerular filtration rate; IMT, intima-media thickness.
