# Supplementary material for: Serum cystatin C is an independent biomarker associated with the renal resistive index in patients with chronic kidney disease
Source: PLoS One. 2018 Mar 7;13(3):e0193695. doi: 10.1371/journal.pone.0193695 (PMC5841772; doi:10.1371/journal.pone.0193695)
Supplement: S4 Table — (DOCX) [file pone.0193695.s004.docx]

**S4 Table. The multivariate odds ratios (95% CI) for baPWV≧1400.**

| Parameter | OR (95% CI) | P-value |
| --- | --- | --- |
| Age (per 10 years) | 3.24 (1.80-6.91) | 0.0005 |
| Gender | 1.07 (0.49-2.30) | 0.8580 |
| SBP (per 10mmHg) | 1.13 (0.54-2.41) | 0.7385 |
| DBP (per 10mmHg) | 1.84 (0.72-5.14) | 0.2167 |
| eGFR (per 10mL/min/1.73m^2^) | 0.82 (0.53-1.22) | 0.3327 |
| Albuminuria (per 500mg/day) | 0.82 (0.59-1.14) | 0.1918 |
| Cystatin C (per 0.5mg/L) | 1.13 (0.69-1.90) | 0.6317 |

DBP, diastolic blood pressure; eGFR, estimated glomerular filtration rate; SBP, systolic blood pressure.
