# Supplementary material for: Serum cystatin C is an independent biomarker associated with the renal resistive index in patients with chronic kidney disease
Source: PLoS One. 2018 Mar 7;13(3):e0193695. doi: 10.1371/journal.pone.0193695 (PMC5841772; doi:10.1371/journal.pone.0193695)
Supplement: S5 Table — (DOCX) [file pone.0193695.s005.docx]

**S5 Table. The multivariate odds ratios (95% CI) for Max IMT≧1.1.**

| Parameter | OR (95% CI) | P-value |
| --- | --- | --- |
| Age (per 10 years) | 8.81 (3.10-38.21) | 0.0006 |
| Gender | 0.83 (0.37-1.83) | 0.6374 |
| SBP (per 10mmHg) | 0.91 (0.47-1.75) | 0.7639 |
| DBP (per 10mmHg) | 2.31 (0.87-6.93) | 0.1052 |
| eGFR (per 10mL/min/1.73m^2^) | 1.05 (0.60-1.80) | 0.8699 |
| Albuminuria (per 500mg/day) | 0.90 (0.63-1.20) | 0.5087 |
| Cystatin C (per 0.5mg/L) | 1.09 (0.61-1.99) | 0.7657 |

DBP, diastolic blood pressure; eGFR, estimated glomerular filtration rate; IMT, intima-media thickness; SBP, systolic blood pressure.
