# Supplementary figures and images for: Serum cystatin C is an independent biomarker associated with the renal resistive index in patients with chronic kidney disease
Source: PLoS One. 2018 Mar 7;13(3):e0193695. doi: 10.1371/journal.pone.0193695 (PMC5841772; doi:10.1371/journal.pone.0193695)

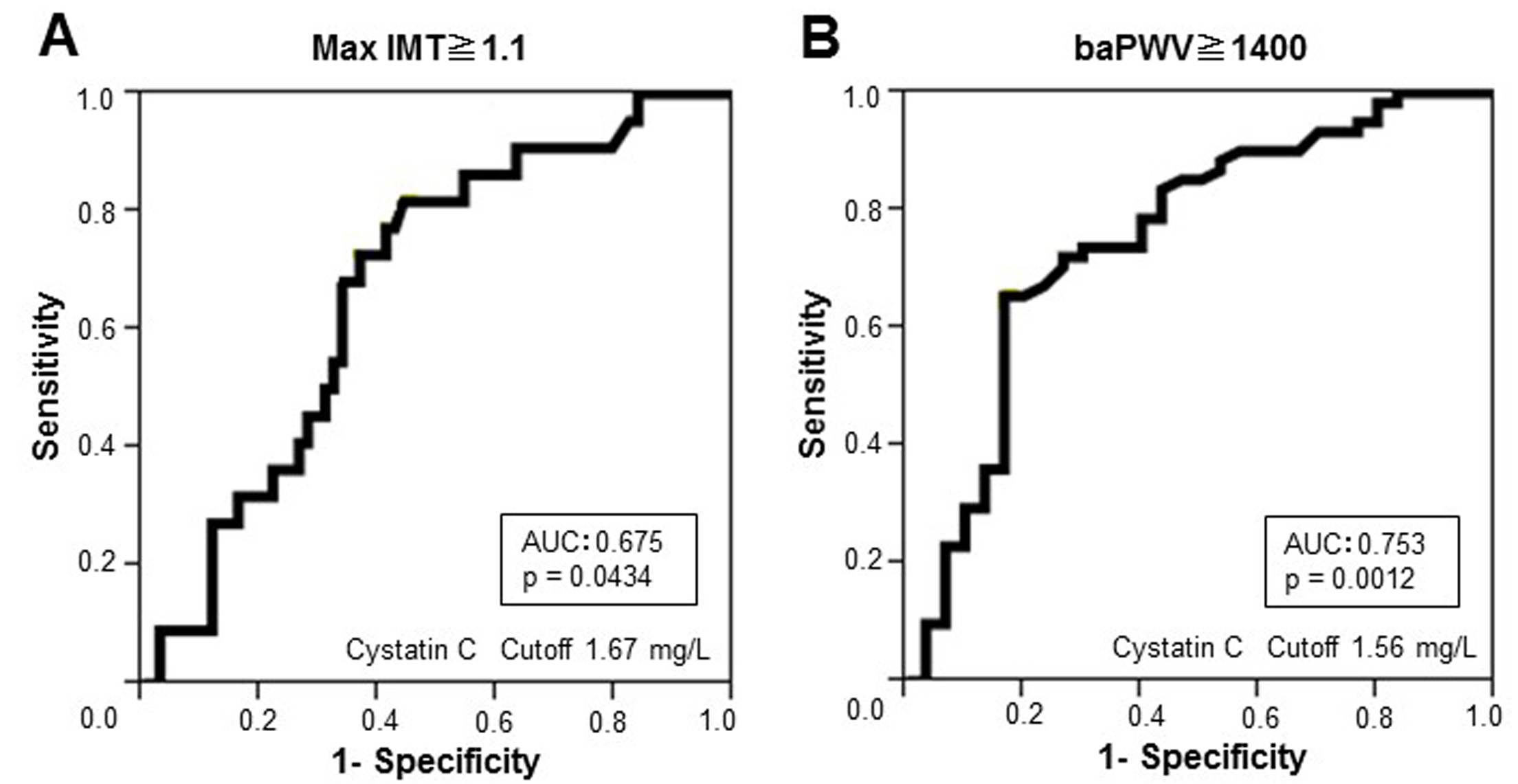

Supplement: S1 Fig — The ROC curves comparing the sensitivity and specificity of cystatin C for predicting a maximum intima-media thickness (IMT) of 1.1 (A) and an ankle-brachial pulse wave velocity (baPWV) of 1400 (B). The AUC values for the ROC curves when cystatin C was used to detect a maximum IMT of 1.1 and a baPWV of 1400, were 0.675 (p = 0.0434) and 0.753 (p = 0.0012), respectively. (TIF) [file pone.0193695.s006.tif]
